# Revisiting the role of bone grafting in scaphoid fixation with volar plates: a multivariable analysis

**DOI:** 10.1007/s12306-025-00926-5

**Published:** 2025-09-27

**Authors:** Salvador J. Gomez Bermudez, Jaime A. Londoño Restrepo, Miguel A. Gómez Trillos, Laura X. Ramírez Carmona, Rafael F. Galindo Zuluaga, Ruben D. Arias Perez, Daniela Carmona Cano, Sebastian Calle Diaz, Santiago Tobon Orrego

**Affiliations:** 1 Hospital Pablo Tobon Uribe, Medellín, Colombia; 2 Pontifical Bolivarian University, Medellín, Colombia; 3 Hospital General de Medellín, Medellín, Colombia; 4 University of Antioquia, Medellín, Colombia

**Keywords:** Scaphoid Fracture, Nounion, Bone Transplantation, Anatomical plate, Fracture Fixation

## Abstract

**Purpose:**

To evaluate the radiographic and functional outcomes of scaphoid fractures and nonunions treated with volar locked plate fixation, with or without autologous bone grafting, and to explore whether graft use was associated with improved consolidation or function.

**Methods:**

This retrospective cohort study included 19 adult patients who underwent surgical treatment with volar anatomical plates for scaphoid fractures or nonunions. Radiographic union was assessed at 3, 6, and 12 months. Functional outcomes were measured using the QuickDASH score preoperatively and at 12 months postoperatively. Graft use was determined intraoperatively based on defect characteristics. Statistical analyses included non-parametric tests and multivariable models.

**Results:**

The mean patient age was 24.5 ± 5.4 years, and 94.7% were male. Scaphoid nonunion was present in 11 patients (57.9%), and autologous bone grafting was performed in 15 (78.9%). Radiographic consolidation was achieved in 94.7% of cases at 12 months. QuickDASH scores improved significantly (mean change: 20.6 points; *p* < 0.001). There were no significant differences in union or functional outcomes between grafted and non-grafted patients (*p* = 1.000 and *p* = 0.115, respectively). Interestingly, patients with nonunions demonstrated significantly better postoperative function than those with acute fractures (*p* = 0.034), although this did not exceed the minimal clinically important difference. Multivariable analysis failed to identify predictors of union or function, explaining only 37% of the variance.

**Conclusion:**

Volar locked plate fixation provides high union rates and significant functional improvement in scaphoid fractures and nonunions. Bone grafting did not confer additional benefit, supporting selective rather than routine use. Further studies are warranted to clarify prognostic factors and optimize treatment strategies.

## Introduction

Scaphoid fractures are the most common carpal bone injury, accounting for up to 70% of all carpal fractures and approximately 2 to 7% of all skeletal fractures in the general population. Their estimated annual incidence is 29 cases per 100,000 individuals [1]. These fractures primarily affect young, active individuals, particularly males, due to their frequent involvement in high-energy trauma or sports-related activities [1]. The scaphoid’s unique anatomical and vascular characteristics, bridging the proximal and distal carpal rows and relying predominantly on retrograde intraosseous blood flow, render it particularly vulnerable to delayed healing, nonunion, and avascular necrosis (AVN), particularly when diagnosis or stabilization is delayed [2]

While nondisplaced fractures often respond well to conservative treatment, displaced, comminuted, or unstable fractures, as well as chronic nonunions, frequently necessitate surgical intervention [3]. Failure to achieve timely union can result in serious sequelae, including scaphoid nonunion advanced collapse, progressive carpal collapse, degenerative arthritis, and chronic pain [4]

Traditional surgical techniques for scaphoid fixation have relied heavily on headless compression screws, often in combination with autologous bone grafts [5]. These techniques have yielded good results in select cases, with union rates typically ranging from 80 to 90% [6]. Although this approach yields good union rates in simple cases, it may be biomechanically insufficient in complex scenarios involving comminution, proximal pole involvement, or bone defects, the limited rotational and axial stability offered by single-screw constructs may be insufficient [5, 7].

In response to these limitations, volar locking plate fixation has emerged as a promising alternative. Volar plates provide angular stability, better fragment control, and enhanced mechanical resistance to shear and torsional forces, advantages that are particularly beneficial in unstable or segmental fractures [8]. Biomechanical studies support the superiority of volar plates over screw fixation in restoring carpal architecture and facilitating early mobilization [5, 8]. Clinical series have shown union rates exceeding 95%, even in previously failed or recalcitrant cases [9].

Autologous bone grafting remains a cornerstone in the management of segmental loss or biologically compromised bone. However, the necessity of grafting in all cases treated with volar plates remains controversial. Some authors have reported excellent outcomes without bone grafting in selected cases with stable fixation and minimal defects [6, 10], prompting a shift toward individualized grafting strategies.

Despite these promising results, evidence regarding the selective use of bone grafts in conjunction with volar plating remains limited, particularly in complex fractures or chronic nonunions. Moreover, few studies have employed multivariable modeling to identify independent predictors of radiographic union or functional recovery in this population, limiting our ability to tailor treatment decisions based on patient or fracture characteristics.

The objective of this study was to evaluate the radiographic and functional outcomes of scaphoid fractures and nonunions treated with volar locked plate fixation, with or without autologous bone grafting. Additionally, we sought to determine whether graft use or other factors were independently associated with improved healing or functional outcomes.

## Materials and methods

### Study design and patient selection

This was a retrospective cohort study of patients with acute scaphoid fractures or radiographic nonunion, all of whom were treated surgically with locked anatomical plates at a high-volume trauma referral center between 2020 and 2023. The decision to use autologous bone grafting was made intraoperatively at the discretion of the lead surgeon. Grafting was indicated in cases where the fracture fragments exhibited suboptimal bone-to-bone contact after reduction, or when voids or cortical defects were evident that could compromise mechanical stability or biological healing. All procedures were performed by three orthopedic surgeons with fellowship training in hand surgery.

The study included patients over 18 years of age. Patients with a history of autoimmune inflammatory disease or documented carpal infection at the time of surgery were excluded. A non-probabilistic, consecutive sampling method was used, including all eligible patients treated during the study period without randomization. Initially, 25 patients were assessed; two were excluded based on inclusion/exclusion criteria and four were lost to follow-up. A total of 19 patients completed the study and met all eligibility and follow-up requirements.

### Radiographic and clinical assessment

All patients underwent standardized wrist radiographs, including posteroanterior (PA), PA in ulnar deviation, lateral, and oblique views, preoperatively and at 3, 6, and 12 months postoperatively. Preoperative computed tomography (CT) scans were obtained in all cases to characterize fracture patterns and plan surgical treatment. The location of the fracture was classified as proximal pole, waist, or distal pole.

Radiographic consolidation was assessed independently by two orthopedic surgeons who were not involved in the surgical procedures. Consolidation was defined as bridging trabeculae across the fracture site in at least three of four radiographic views. Discrepancies were resolved by consensus. Follow-up CT scans were not routinely performed.

Demographic data (age, sex, laterality) and surgical details (use and location of autologous bone graft) were recorded. Monitored complications included infection, neurovascular injury, and hardware failure. Functional outcome was assessed using the QuickDASH clinical score preoperatively and at 12 months postoperatively. The presence of pain after radiological consolidation was recorded as a binary variable (yes/no). All assessments were performed following a standardized protocol.

### Statistical analysis

Statistical analyses were performed using Python (v3.11). The SciPy and Statsmodels libraries were used for statistical testing, Pandas for data management, Seaborn and Matplotlib for data visualization, and scikit-learn for fitting penalized logistic regression models.

Quantitative variables were described using mean and standard deviation ( ±) or median and interquartile range (IQR). The distribution was assessed by the Shapiro–Wilk test. Since most variables were non-normally distributed, non-parametric tests were used for inferential analysis.

The Wilcoxon signed-rank test was used for paired comparisons, and the Mann–Whitney U test was used for unpaired data for comparisons between two groups, and the Kruskal–Wallis test for comparisons involving more than two groups, followed by Bonferroni-adjusted post hoc tests when appropriate. Associations between categorical variables were assessed using Fisher’s exact test or the chi-squared test, and odds ratios (OR) with 95% confidence intervals (CI) were reported when applicable. Spearman’s rank correlation coefficient was used to evaluate associations between continuous variables. A two-tailed *p* value < 0.05 was considered statistically significant throughout the analysis.

Penalized logistic regression models (Ridge) with internal cross-validation were used to identify independent predictors of radiographic consolidation at 12 months. Clinical, demographic, and surgical variables were included as predictors. Models reported standardized coefficients and estimated OR, interpreted from a clinical perspective.

Additionally, a multiple linear regression model was fitted to identify independent predictors of postoperative functional outcome, measured using the QuickDASH score at 12 months. Clinical, demographic, and surgical variables were included as predictors. Coefficients, 95% confidence intervals, and *p*-values were reported for each variable. The coefficient of determination (R^2^) and adjusted R^2^ were used to evaluate the explanatory power of the model.

## Results

Nineteen patients underwent volar locked plate fixation for scaphoid fractures or nonunions. The mean age was 24.5 ± 5.4 years, and 94.7% were male. Nonunion was diagnosed in 11 patients (57.9%) and acute fracture in 8 (42.1%). Most fractures involved the waist (57.9%), followed by the distal pole (31.6%) and proximal pole (10.5%), (Table 1).

**Table 1:** Patient demographics and clinical characteristics

Variable	Value
Age (years), mean ± SD	24.5 ± 5.4
Sex, n (%) male	18 (94.7%)
Hand dominance, n (%) right-handed	18 (94.7%)
Fracture on dominant hand, n (%)	9 (47.4%)
Autologous graft used, n (%)	15 (78.9%)
QuickDASH preoperative, mean ± SD	26.9 ± 13.7
QuickDASH postoperative, mean ± SD	6.3 ± 6.6
Residual pain after consolidation, n (%)	10 (52.6%)
Implant removal required, n (%)	9 (47.4%)
Infection, n (%)	1 (5.3%)

Autologous bone grafting was performed in 15 patients (78.9%), predominantly harvested from the ipsilateral distal radius. Radiographic union was achieved in 8 patients (42.1%) by 3 months, 15 (78.9%) by 6 months, and 18 (94.7%) by 12 months. Only one case failed to consolidate.

**Fig. 1:**
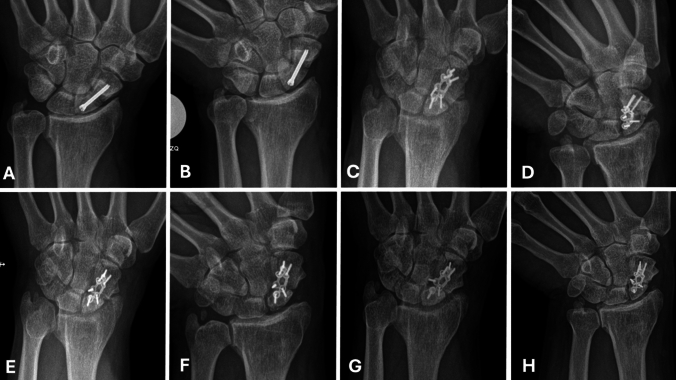
Radiographic sequence of the only patient who failed to achieve union at 12-month follow-up. **A**. Posteroanterior (PA) view of the left wrist showing scaphoid nonunion diagnosed 48 months after the initial surgery. **B**. PA view with ulnar deviation. **C**. PA view at 3 months following volar locked plate fixation. **D**. PA view with ulnar deviation at 3 months. **E**. PA view at 6 months postoperatively. **F**. PA view with ulnar deviation at 6 months. **G**. PA view at 12 months of follow-up. **H**. PA view with ulnar deviation at 12 months, showing persistent radiographic signs of nonunion

**Fig. 2:**
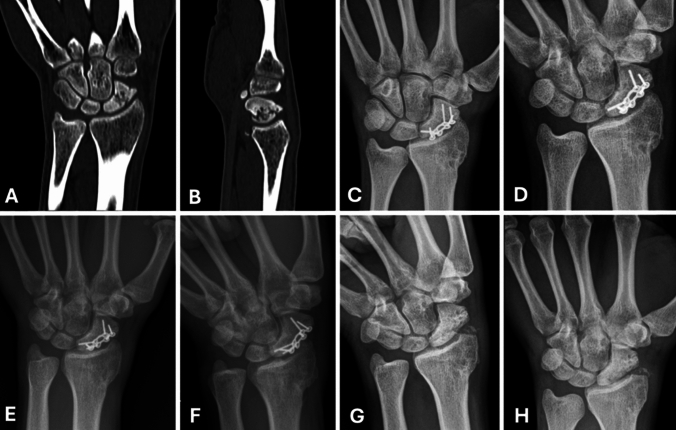
Radiological sequence of a patient who suffered acute trauma, with a scaphoid waist fracture treated with volar plate fixation. **A**. Coronal view of a preoperative computed tomography (CT) scan of the right wrist. **B**. Sagittal CT view showing a displaced scaphoid waist fracture. **C**. Posteroanterior (PA) radiograph at 3 months postoperatively. **D**. PA view with ulnar deviation at 3 months, demonstrating radiographic signs of bone consolidation. **E**. PA view at 6 months of follow-up. **F**. PA view with ulnar deviation at 6 months, confirming continued radiographic consolidation. **G**. PA view with ulnar deviation at 12 months of follow-up. **H**. PA view at 12 months, after Implant removal (performed at 11 months), with maintained bone union

At the 3-month follow-up, radiographic consolidation was observed in 8 patients (42.1%). By 6 months, the cumulative consolidation rate increased to 15 patients (78.9%), and by 12 months, 18 out of 19 patients (94.7%) had achieved radiographic healing, (Fig. 1 and 2).

**Fig. 3:**
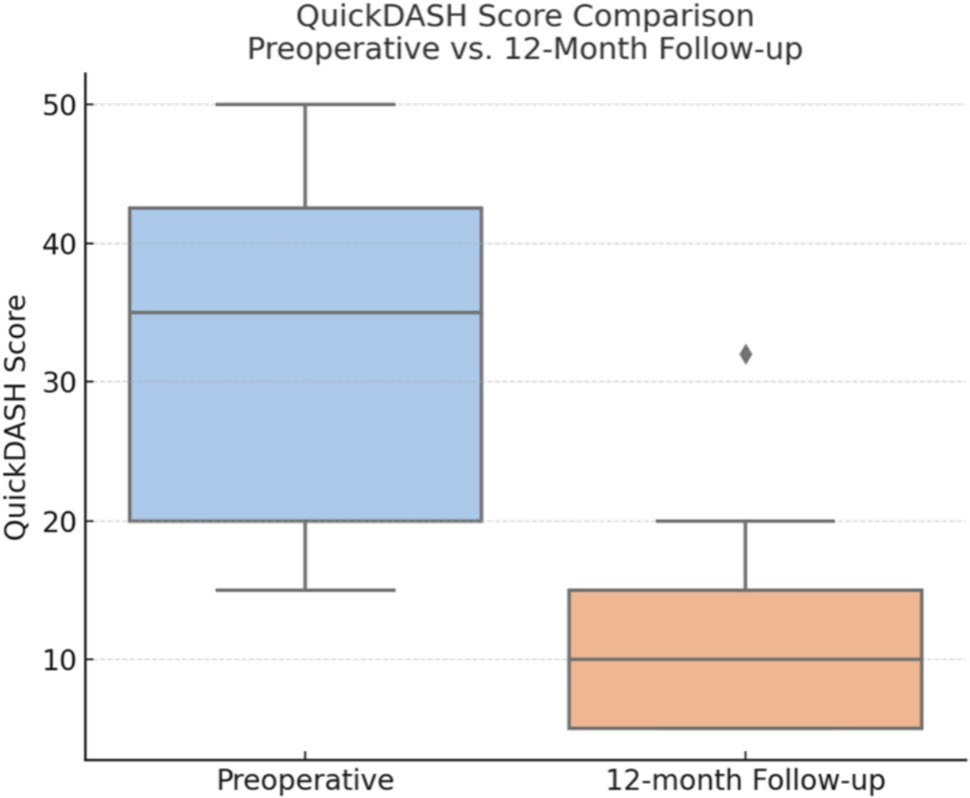
Comparison of preoperative and postoperative QuickDASH scoring results

Regarding functional outcomes assessed with the QuickDASH score, a mean improvement of 20.6 points was observed between the preoperative evaluation and the 12-month follow-up. This difference was statistically significant (*p* < 0.001), with a 95% CI of 16.7 to 24.5 points, (Fig. 3).

The presence of pain after radiological consolidation was reported in 10 patients (52.6%), secondary to which Implant removal was required in 9 cases (47.4%) during follow-up. One case of superficial infection was documented (5.3%), and no hardware failures were observed in any patient.

Patients who did not receive a graft had a slightly higher mean QuickDASH score at 12 months (mean difference = 4.45 points), although this difference did not reach statistical significance (*p* = 0.115). Regarding radiographic consolidation, no association was observed with graft use (*p* = 1.000).

**Fig. 4:**
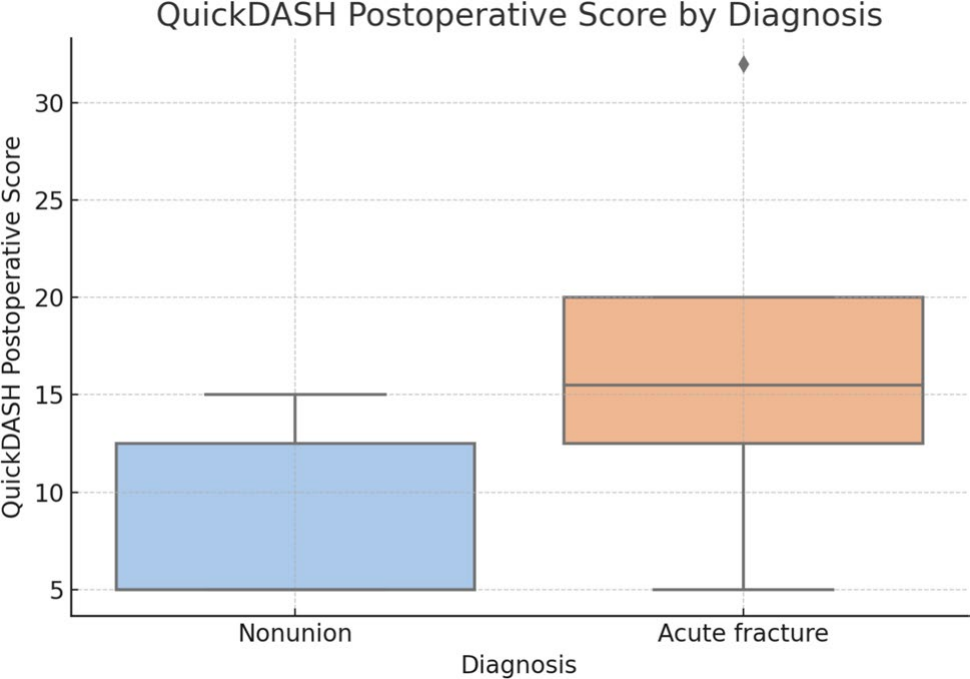
Comparison of the results of the QuickDASH postoperative score between nonunion and acute fracture patients

Patients with an initial diagnosis of nonunion showed significantly better postoperative functional outcomes compared to those with acute fractures, as measured by the QuickDASH score. The mean QuickDASH score at 12 months was 8.6 in the nonunion group and 16.0 in the acute fracture group, yielding a mean difference of − 7.4 points. This difference was statistically significant (*p* = 0.0337), with a 95% CI ranging from − 14.0 to 0.8 points, (Fig. 4).

No correlation was found between patient age and the postoperative QuickDASH score (*p* = 0.9127). A penalized logistic regression model using L2 regularization (Ridge) was fitted to identify independent predictors of radiographic consolidation at 12 months. None of the variables included in the model demonstrated a clinically meaningful or statistically significant association with radiographic healing, (Table 2).

**Table 2:** Penalized logistic regression model (Ridge) for 12-month consolidation

Variable	Coefficient (β)	OR (exp β)
Age	+ 0.0002	1.0002
Graft	+ 0.0002	1.0002
Fracture location	+ 0.0001	1.0001
Diagnosis	− 0.0000	1.0000
Infection	+ 0.0000	1.0000

A multiple linear regression model was developed to evaluate independent predictors of postoperative functional outcomes, measured by the QuickDASH score at 12 months. The model explained 37.1% of the variance in the QuickDASH score (R^2^ = 0.3705). Nonunion diagnosis showed a trend toward improved functional outcomes compared to acute fractures (β =  + 9.18, *p* = 0.069), though this did not reach statistical significance. Other predictors were not significantly associated with functional recovery the postoperative QuickDASH score, (Table 3).

**Table 3:** Multiple linear regression model (QuickDASH postoperative score)

Variable	Coef. (β)	Standard Error	p-value	95% CI (Lower)	95% CI (Upper)
Age	+ 0.45	0.38	0.263	− 0.39	+ 1.30
Graft	− 3.02	3.82	0.446	− 11.42	+ 5.38
Fracture location	− 1.24	3.87	0.754	− 9.76	+ 7.27
Diagnosis	+ 9.18	4.55	0.069	− 0.84	+ 19.20
Infection	+ 3.95	6.27	0.540	− 9.43	+ 17.33
Postoperative pain	+ 5.94	4.74	0.232	− 5.07	+ 16.96
Plate removal	− 3.71	4.57	0.418	− 13.66	+ 6.25
Intercept	+ 9.48	32.96	0.779	− 63.07	+ 82.04

## Discussion

The significant improvement in functional outcomes observed in our cohort, as measured by the QuickDASH score, highlights the potential efficacy of locked plate fixation for treating both acute scaphoid fractures and nonunions. The mean improvement of 20.6 points was statistically significant (*p* < 0.001), exceeding the minimal clinically important difference (MCID) reported for this score in upper limb pathology [11, 12]. This finding is consistent with previous studies demonstrating the effectiveness of surgical fixation in restoring function and reducing disability in scaphoid pathology [4, 13, 14].

Interestingly, patients with nonunion achieved better functional scores than those with acute fractures, a finding that may appear counterintuitive. This could be explained by stricter surgical indications, longer adaptation periods, or more structured rehabilitation protocols in the nonunion group. Although statistically significant (*p* = 0.034), the difference did not reach the MCID threshold [11, 12], suggesting similar overall recovery in both groups.

Radiographic consolidation was achieved in 94.7% of patients by 12 months, with no hardware failures and a very low infection rate. However, our penalized logistic regression model failed to identify any statistically or clinically meaningful predictors of consolidation. All odds ratios were approximately 1.000, suggesting that factors such as age, graft use, fracture site, diagnosis, or infection did not independently influence healing outcomes in this sample (See Table 2). This likely reflects the limited variability in the outcome (only one non-consolidated case), and highlights a key limitation of predictive modeling in small surgical series.

Multivariable analysis did not identify independent predictors of union or functional recovery. The penalized logistic regression model for consolidation yielded odds ratios close to 1.000 for all variables, likely due to the low event rate (only one nonunion case). The linear regression model explained only 37% of the variation in QuickDASH scores. The absence of significant predictors is likely due to the low event rate and small sample size, which constrain the statistical power of multivariable modeling, rather than a true lack of associations. Larger studies are required to validate these findings.

Importantly, nearly half of the cohort (47%) required plate removal due to symptomatic hardware, consistent with other reports documenting mechanical irritation, impingement, or restricted range of motion following volar plate fixation [15, 16]. This complication, while not catastrophic, reinforces the need for precise implant contouring and preoperative counseling regarding potential secondary procedures, particularly in active or younger patients with high functional demands [17, 18].

In our cohort, the selective use of autologous bone grafting did not result in significant differences in radiographic union or functional outcomes compared to cases treated without grafts. Although grafted patients showed a modest trend toward improved QuickDASH scores at 12 months, the difference was not statistically nor clinically significant [11, 12]. These findings support emerging evidence that, when stable fixation and adequate fragment apposition are achieved, bone grafting may not be universally necessary, particularly in non-comminuted fractures with minimal bone loss [6].

Prior studies have similarly demonstrated high union rates using volar plate fixation alone, suggesting that the mechanical stability provided by locked plates may compensate for the absence of graft in selected cases [9, 10]. Our results reinforce the concept that bone grafting should not be routine, but rather guided by specific intraoperative findings such as poor contact, or compromised biology. This tailored approach may reduce unnecessary graft harvesting, especially in young, active patients.

This study has several limitations. The retrospective design, lack of a randomized control group, and small sample size inherently limit the generalizability and statistical power of the findings. The decision to graft was not randomized but based on intraoperative judgment, which may introduce selection bias. Although multivariable modeling was used to mitigate confounding, its power was limited by the sample size and low variability in outcomes.

In conclusion, volar locking plate fixation, when combined with selective and individualized bone grafting, appears to be a biomechanically and clinically effective strategy for managing complex scaphoid fractures and nonunions. In selected cases without significant bone loss or instability, stable plate fixation alone may be sufficient to achieve union and restore function. Although patients with nonunions demonstrated better postoperative QuickDASH scores compared to those with acute fractures, this difference was not clinically meaningful. The lack of significant predictors of radiographic or functional outcomes, likely due to sample size limitations, highlights the complexity of prognosis in this population. Additionally, the relatively high rate of symptomatic hardware requiring removal underscores the importance of careful implant selection and patient counseling. Further prospective, adequately powered studies are warranted to optimize patient selection and treatment protocols.

## Data Availability

No datasets were generated or analysed during the current study.
